# Impact of postoperative complications and type 2 diabetes on breast cancer recurrence and mortality

**DOI:** 10.1093/bjs/znaf176

**Published:** 2025-08-23

**Authors:** Kasper A Kjærgaard, Peer Christiansen, Signe Borgquist, Deirdre Cronin-Fenton

**Affiliations:** Department of Clinical Epidemiology, Department of Clinical Medicine, Aarhus University Hospital, Aarhus University, Aarhus, Denmark; Department of Oncology, Department of Clinical Medicine, Aarhus University Hospital, Aarhus University, Aarhus, Denmark; Department of Plastic and Breast Surgery, Department of Clinical Medicine, Aarhus University Hospital, Aarhus University, Aarhus, Denmark; Department of Oncology, Department of Clinical Medicine, Aarhus University Hospital, Aarhus University, Aarhus, Denmark; Department of Oncology, Clinical Sciences, Lund University, Lund, Sweden; Department of Clinical Epidemiology, Department of Clinical Medicine, Aarhus University Hospital, Aarhus University, Aarhus, Denmark

## Abstract

**Background:**

Type 2 diabetes (T2D) is associated with increased risk of complications after breast cancer surgery. This study investigated systemic and surgical-site postoperative complications and the risk of early and late breast cancer recurrence and mortality, overall, and according to T2D status.

**Methods:**

From the Danish Breast Cancer Group (DBCG) and Danish registries, a cohort of women with incident early-stage breast cancer diagnosed 1996–2017 was assembled. All women underwent mastectomy or breast-conserving surgery. Using diagnostic codes, systemic and surgical-site complications within 30 days of surgery were defined. Early recurrence (<10 years from diagnosis) was ascertained from the DBCG and late recurrence (≥10 years after diagnosis) via a validated registry-based algorithm. Incidence rates (IRs) per 1000 person-years were calculated, and Cox regression was used to estimate adjusted hazard ratios (aHRs) of early and late recurrence, and death, according to postoperative complications. Potential effect measure modification by T2D was evaluated.

**Results:**

Among 58 198 women undergoing breast cancer surgery, 6285 (10.8%) had postoperative complications. Overall, 747 and 5756 women with and without complications developed early recurrence (IR: 19.7 and 17.9, aHR = 1.04, 95% c.i. 0.96, 1.13), whereas 314 and 3314 women developed late recurrence (IR: 23.9 and 26.0, and aHR = 0.90, 95% c.i. 0.80, 1.02). Prevalent T2D did not impact these findings. Women with postoperative complications had higher mortality rates than those without complications (aHR = 1.11; 95% c.i. 1.06, 1.16); especially those with T2D *versus* no T2D (aHR = 1.50; 95% c.i. 1.30, 1.73).

**Conclusion:**

Postoperative complications after breast cancer primary surgery did not impact recurrence risk but had a negative impact on survival, especially in women with T2D.

## Teaser

Whether postoperative complications affect breast cancer prognosis is not clear. In this large population-based cohort study including 58 198 women with early-stage breast cancer, postoperative complications within 30 days of cancer surgery were not associated with increased risk of early or late recurrence. However, mortality rate was higher, particularly among women with type 2 diabetes.

## Introduction

Breast cancer is the most commonly diagnosed cancer worldwide and accounts for nearly 25% of all cancers among women^1^. With improvements in diagnostics and treatment, the proportion of long-term breast cancer survivors is increasing^2^. Surgery, performed as either mastectomy or breast-conserving surgery (BCS), is standard for treatment with curative-intent, but comes with some risks. In cancer surgery, surgical site complications account for more than one-third of all complications. Such complications have been associated with an increased risk of colorectal, head and neck, and gastric cancer recurrence^3^. Postoperative complications may also impact breast cancer prognosis^4^. The mechanisms underlying these associations remain unclear. They may include physiological stress and systemic inflammation following surgery and anaesthesia^5,6^, promoting angiogenesis and the growth and spread of residual tumour cells^5,6^. Postoperative complications could delay subsequent adjuvant treatment^4,7,8^. Yet, observational studies investigating the potential impact of postoperative complications on breast cancer outcomes are sparse and have limitations. Many have focused on mastectomy with immediate breast reconstruction and/or surgical site complications^9–12^, with only limited focus on BCS^13,14^ and systemic complications^15^.

Type 2 diabetes (T2D) at diagnosis is associated with poorer breast cancer prognosis^16,17^. The authors have previously shown an increased risk of complications (including both surgical site and systemic complications such as infection, bleeding, venous thromboembolism, arterial cardiovascular disease, and kidney disease) following primary breast cancer surgery in women with T2D compared with those without^18^. It is not known if and how such systemic postoperative complications influence breast cancer prognosis. Furthermore, the impact of T2D on the risk of recurrence and mortality in women who suffer from postoperative complications has not been evaluated.

This nationwide population-based cohort study was therefore conducted to investigate the association between any complication, systemic or surgical site, occurring within 30 days of primary breast cancer surgery and the risk of recurrence and mortality among women with early-stage breast cancer. The potential synergistic effects between complications and T2D were evaluated on the incidence rates of early and late breast cancer recurrence and mortality.

## Methods

### Electronic data collection

The Danish National Health Service enables all Danish residents to have tax-funded and unrestricted access to healthcare services encompassing public hospitals and general practitioners^19^. Utilizing the civil personal registration number, a unique personal identifier allocated to every Danish resident since 1968, linkage of data across several Danish population-based and medical registries is possible. Registries used in the present study included the Danish Breast Cancer Group (DBCG) clinical database^20^, the Danish National Patient Registry (DNPR)^21^, the Danish Cancer Registry (DCR)^22^, the Danish Pathology Registry (DPR)^23^, the Danish Civil Registration System^24^, and the Danish National Prescription Registry^25^.

### Study population

All female residents of Denmark aged at least 18 years with an incident diagnosis of early-stage, defined as stage I–III, breast cancer between 1 January 1996 and 31 May 2017 registered in the nationwide DBCG clinical database were included (*Supplementary Methods*). Women were included if they had been resident in Denmark for at least 6 months prior to surgery and underwent primary breast cancer surgery via mastectomy or BCS. If a patient underwent multiple surgical procedures (including primary breast cancer surgery with mastectomy or BCS, or reoperation due to insufficient margins or subsequent axillary dissection), the date of the definitive procedure was designated as the index date.

### Postoperative complications

ICD-10 codes from the DNPR were used to define the first event of postoperative complications occurring within 30 days following the primary surgical procedure. The complications were identified through at least one hospital readmission or outpatient visit for any of the diagnoses listed in *Table S1*. Complications were stratified by predefined groups into systemic complications (that is a composite of systemic bleeding, infection, venous thromboembolism, arterial cardiovascular disease, and kidney disease) and surgical-site complications (for example wound infections or reoperations because of bleeding at the surgical site or local infection).

### Type 2 diabetes

Prevalent diagnoses for T2D were identified based on diagnostic codes (ICD, eighth and tenth revisions) obtained from the DNPR or the redemption of at least one prescription for glucose-lowering medications documented in the Danish National Prescription Registry using Anatomical Therapeutic Chemical (ATC) classification codes. More information on specific ICD-8 and -10 codes, ATC codes, and the definition of T2D have been outlined elsewhere^18^.

### Breast cancer recurrence and mortality rate

Breast cancer recurrence was categorized as early recurrence (within 10 years of primary breast cancer diagnosis) or late recurrence (10 years or more after initial breast cancer diagnosis). Data on early recurrence were obtained from DBCG and defined as any local, regional, or distant recurrence^26^. Late recurrence was defined via a prespecified validated algorithm incorporating clinical and diagnostic information from DNPR and cancer diagnoses from DNPR, DCR, and DPR, and contralateral breast cancer (CBC) from a CBC database (*Supplementary Methods*)^27^. Due to data availability, the CBC database was not used to define late recurrence in the current study^28^. To verify a diagnosis of late recurrence using the algorithm, the recurrence was confirmed if no new cancer diagnosis was made during the 90-day period after the initial recurrence registration. To be eligible for a late breast cancer recurrence, women had to survive 10 years after their initial breast cancer surgery with no recurrence or second cancer diagnosed in that period.

Information on all-cause mortality rate was obtained through linkage to the Civil Registration System^24^.

### Covariates

Patient characteristics on emigration, vital status, and marital status were retrieved from the Danish Civil Registration System. Marital status at the time of surgery was categorized as married (married or in a partnership) or single (unmarried, divorced, or widowed). Information on the type of primary breast cancer surgery (mastectomy or BCS) with and without radiation therapy, oestrogen-receptor (ER) and human epidermal growth factor receptor 2 (HER2) status, tumour size, lymph node status, adjuvant endocrine therapy, adjuvant and neoadjuvant systemic treatment (chemotherapy with or without anti-HER2 treatment), and menopausal status were collected from the DBCG. Information on UICC stage based on the TNM classification was collected from the DCR. From the DNPR, data on pre-existing co-morbid diseases up to 10 years before initial breast cancer surgery were retrieved. Utilizing a modified Charlson Co-morbidity Index (CCI) score that excludes breast cancer, the severity of baseline co-morbidity was classified into three categories: no co-morbidity (CCI score = 0), low co-morbidity (CCI score = 1–2), or high co-morbidity (CCI score ≥ 3). Information on baseline use of comedications including menopausal hormone therapy, systemic glucocorticoids, lipophilic statins, selective serotonin reuptake inhibitors (SSRI), and aspirin within 1 year prior to breast cancer surgery was obtained from the Danish National Prescription Registry.

### Statistical analyses

Patients were followed from 30 days after their definitive breast cancer surgery as registered in DBCG until a confirmed breast cancer recurrence, emigration, any second cancer, contralateral breast cancer, death, or end of study period (31 December 2022), whichever came first. Crude incidence rates (IRs) per 1000 person-years and associated 95% confidence intervals of early and late breast cancer recurrence according to postoperative complications were computed. Cumulative incidence functions of early and late recurrence were plotted, considering death as a competing risk. The probability of death from any cause was computed and visualized using the Kaplan–Meier estimator. The interaction effect of complications and T2D on the IR of recurrence was examined by calculating interaction contrasts^29^. The interaction contrast is described as a metric that quantifies the increase or decrease in the IR beyond what can be attributed to the baseline recurrence rate following breast cancer surgery, the impact of complications on the IR, and the influence of T2D on the IR (for example of interaction contrasts calculations, see *Supplementary Methods*)^30^. Cox proportional hazard regression models were used to evaluate the association of any postoperative complication with breast cancer recurrence, adjusting for potential confounders. Potential confounder that were adjusted for were age, year of surgery, co-morbidity score, UICC stage, type of surgical procedure, neoadjuvant systemic therapy, and baseline use of relevant comedications such as lipophilic statins, aspirins, and SSRI. Directed-acyclic graphs (DAGs) were used to identify the relevant set of covariates for the adjusted model. Proportionality of hazards were inspected with no violations detected. The analyses of both early and late recurrence were stratified by age group, calendar period, co-morbidity, cancer stage, ER status, neoadjuvant therapy, type of surgery, and the systemic or surgical-site complication groups.

A prespecified sensitivity analysis was conducted, changing the 30-day window for postoperative complications to start follow-up at 14 days after the date of definitive breast cancer surgery.

## Results

The cohort included 58 198 women, of whom 6285 (10.8%) developed a postoperative complication within 30 days of primary surgery. Women with postoperative complications were more likely to be 65 years or older, postmenopausal, single, have more co-morbidities, larger tumours, and node-positive disease (and thus a higher cancer stage). They more frequently underwent mastectomy than BCS. Women with complications were also more often concurrent users of lipophilic statins, aspirins, and SSRIs (*Table 1*).

**Table 1:** Baseline patient, tumour, and treatment characteristics of women diagnosed with early-stage, operable breast cancer in Denmark between 1996 and 2017, according to postoperative complications

Characteristics	All patients *n* = 58 198		Women with T2D *n* = 2923	
	Complication	No complication	Complication	No complication
	(*n* = 6285)	(*n* = 51 913)	(*n* = 483)	(*n* = 2440)
**Patient characteristics**				
Age at index (years), median	61 (52–69)	60 (51–67)	67 (62–74)	66 (60–72)
Age (years)				
≤44	566 (9)	5664 (11)	<5 (1)	47 (2)
45–54	1372 (22)	12 549 (24)	Masked (6)	250 (10)
55–64	1839 (29)	16 389 (32)	159 (31)	724 (30)
65–74	1758 (28)	12 995 (25)	186 (39)	985 (40)
≥75	750 (12)	4315 (8)	112 (23)	434 (18)
Year of breast cancer diagnosis				
1996–2003	1693 (27)	15 183 (29)	67 (14)	429 (18)
2004–2010	2234 (36)	18 404 (36)	176 (36)	882 (36)
2011–2017	2358 (37)	18 326 (35)	240 (50)	1129 (46)
Menopausal status				
Premenopausal	1461 (23)	13 860 (27)	Masked (4)	180 (7)
Postmenopausal	4814 (77)	37 964 (73)	462 (96)	2255 (93)
Missing	11	89	<5	5
Charlson Co-morbidity Index score				
No (0)	4913 (78)	44 974 (87)	261 (54)	1712 (70)
Low (1–2)	1212 (19)	6495 (12)	180 (37)	643 (26)
High (≥3)	160 (3)	444 (1)	42 (9)	85 (4)
Marital status				
Married	3771 (60)	32 487 (63)	266 (55)	1269 (52)
Single	2514 (40)	19 426 (37)	217 (45)	1171 (48)
**Tumour and treatment characteristics**				
UICC stage*				
I	2321 (38)	23 265 (46)	168 (36)	1019 (43)
II	2959 (48)	21 526 (42)	225 (48)	1054 (44)
III	854 (14)	5938 (12)	76 (16)	304 (13)
Missing	115	924	12	49
Tumour size (mm)				
≤20 mm	3660 (60)	33 349 (66)	248 (53)	1465 (61)
>20 mm	2446 (40)	17 188 (34)	218 (47)	918 (39)
Missing	179	1376	17	57
Lymph node status				
Negative	3300 (54)	31 172 (61)	249 (53)	1461 (61)
1–3	1996 (32)	13 874 (27)	141 (30)	613 (26)
4–9	563 (9)	3966 (8)	43 (9)	190 (8)
10+	319 (5)	2108 (4)	37 (9)	124 (5)
Missing	107	793	13	52
ER/ET status				
ER−/ET−	571 (11)	4393 (11)	47 (11)	252 (12)
ER+/ET+	4047 (81)	30 660 (77)	351 (81)	1573 (75)
ER−/ET+	24 (0)	130 (0)	<5 (0)	<5 (0)
ER+/ET−	380 (8)	4783 (12)	Masked (8)	Masked (13)
Type of surgery**				
Mastectomy	2180 (35)	14 670 (29)	178 (38)	704 (30)
Mastectomy + RT	1024 (17)	6843 (14)	60 (13)	252 (11)
BCS + RT	2936 (48)	28 983 (57)	233 (49)	1414 (59)
Missing	145	1417	12	69
Adjuvant chemotherapy	2510 (54)	19 836 (53)	153 (39)	689 (35)
Neoadjuvant systemic therapy	130 (2)	983 (2)	14 (3)	48 (2)
Comedications at baseline				
Menopausal hormone therapy	1538 (24)	12 448 (24)	77 (16)	481 (20)
Systemic glucocorticoids	374 (6)	2443 (5)	32 (7)	154 (6)
Lipophilic statins	1063 (17)	6175 (12)	271 (56)	1352 (55)
Non-aspirin NSAIDs	1588 (25)	11 722 (23)	139 (29)	686 (28)
Aspirin (low and high doses)	964 (15)	4719 (9)	203 (42)	836 (34)
SSRI	604 (10)	3750 (7)	71 (15)	300 (12)

Values are *n* (%) unless otherwise indicated. Abbreviations: T2D, type 2 diabetes, BCS, breast-conserving surgery; ER, estrogen receptor; ET, endocrine therapy; NSAIDs, non-steroidal anti-inflammatory drugs; RT, radiation therapy; SSRI, selective serotonin reuptake inhibitors; UICC, Union for International Cancer Control. *Patients coded with stage 4 breast cancer (*n* = 296 from the overall cohort) have been removed due to possibility of error as these patients per definition should not have been included in the original database. **Missing values in this category are exclusively due to missing information on ITT radiation therapy.

### Early and late breast cancer recurrence

**Fig. 1:**
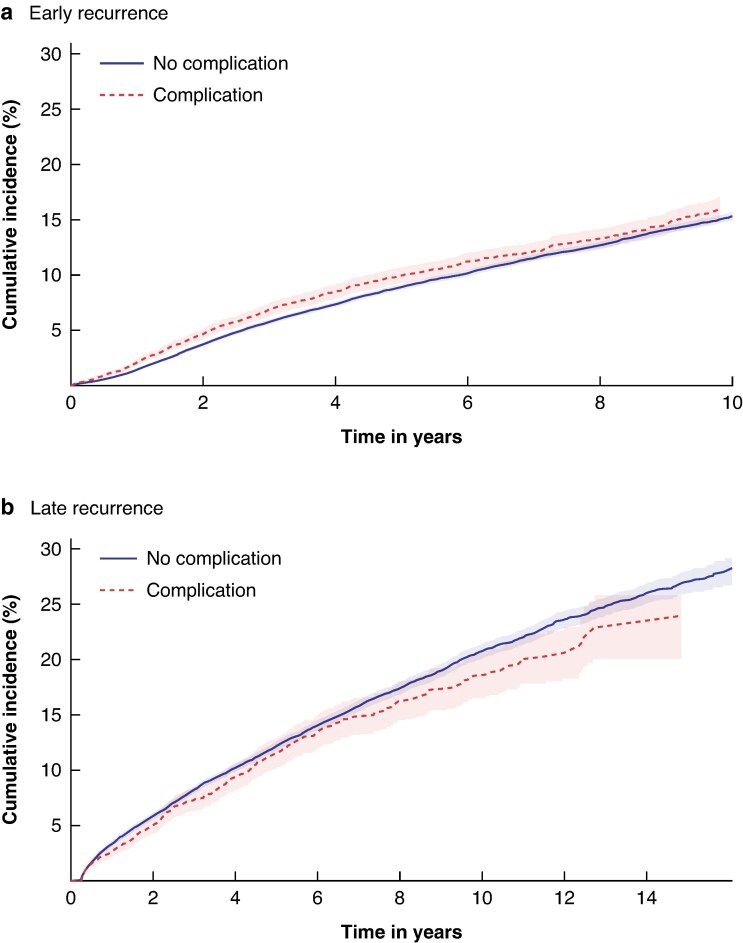
Cumulative incidence of early (a) and late (b) breast cancer recurrence among women with or without a postoperative complication following primary breast cancer surgery during 1997–2017 For panel (**b**), time zero corresponds to 10 years after initial breast cancer diagnosis.

In total, 747 and 5756 women with and without postoperative complications developed an early recurrence, corresponding to IRs of 19.7 (95% c.i. 18.4, 21.2) and 17.9 (95% c.i. 17.4, 18.4) per 1000 person-years (PY) respectively (*Table S2*). The 5-year cumulative incidence of early recurrence was 10.0% and 8.9% for women with and without complications, and 10-year cumulative incidence was 16.0% and 15.3% respectively (*Fig. 1a*). Findings did not support an elevated risk of early breast cancer recurrence among women with postoperative complications (adjusted hazard ratio (aHR) = 1.04, 95% c.i. 0.96, 1.13). Findings were similar in the sensitivity analysis (14-day complication window) (*Table 2*). Systemic complications, a cancer diagnosis in the later calendar period, and age ≥75 years had a minor impact on the risk of early recurrence, but overall, there was no evidence of effect modification in stratified models (*Fig. S1*).

**Table 2:** Early and late breast cancer recurrence, according to postoperative complications and type 2 diabetes in women diagnosed with early-stage breast cancer in Denmark from 1996 to 2017

Complications	Early recurrence				Late recurrence			
	No. of recurrences	Person-years	Crude HR (95% c.i.)	aHR* (95% c.i.)	No. of recurrences	Person-years	Crude HR (95% c.i.)	aHR* (95% c.i.)
**Overall breast cancer cohort**								
Within 30 days								
No complication	5 756	321 802	1.00 (ref)	1.00 (ref)	3 314	127 314	1.00 (ref)	1.00 (ref)
Postoperative complication	747	37 882	1.10 (1.02–1.19)	1.04 (0.97–1.13)	314	13 119	0.92 (0.82–1.03)	0.90 (0.80–1.02)
Within 14 days (*sensitivity analysis)*								
No complication	6 037	334 362	1.00 (ref)	1.00 (ref)	3 439	133 199	1.00 (ref)	1.00 (ref)
Postoperative complication	466	25 322	1.01 (0.92–1.12)	1.02 (0.92–1.12)	189	7 425	0.97 (0.84–1.12)	0.97 (0.84–1.13)
**Women with complications only**								
No type 2 diabetes	694	35 447	1.00 (ref)	1.00 (ref)	306	12 752	1.00 (ref)	1.00 (ref)
Type 2 diabetes	53	2 435	1.08 (0.82–1.43)	1.10 (0.81–1.50)	8	366	0.86 (0.43–1.75)	0.86 (0.42–1.79)

Abbreviation: aHR, adjusted hazard ratio. *Adjusted for age (as a continuous variable), index year, co-morbidity score (no, low, or high), UICC stage (I–III), type of surgery (mastectomy with and without radiation therapy, and BCS), neoadjuvant systemic therapy (yes, no), and baseline use (yes, no) of lipophilic statins, SSRI, and aspirin.

Among 26 193 10-year disease-free survivors, 314 and 3314 women with and without postoperative complications developed late recurrence (IR = 23.9, 95% c.i. 21.4, 26.7 and IR = 26.0, 95% c.i. 25.1, 26.9 per 1000 PY respectively) (*Table S2*). The 5-year cumulative incidence (that is 15 years after primary diagnosis) was 11.5% and 12.2% for women with and without complications, whereas the 10-year cumulative incidence (that is 20 years after primary diagnosis) was 18.5% and 20.8% respectively (*Fig. 1b*). Overall, the aHR for late breast cancer recurrence was 0.90 (95% c.i. 0.80, 1.02) (*Table 2*). Except for a lower risk of late recurrence in women aged 64–74 years, the findings did not vary substantially in the sensitivity or stratified analyses (*Table S2* and *Fig. S1*).

### Mortality rate

**Fig. 2:**
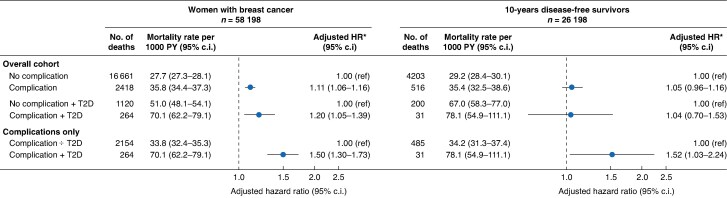
**Mortality rates per 1000 person-years and hazard ratios among women with or without a postoperative complication within 30** **days of primary breast cancer surgery during 1997–2017 and according to type 2 diabetes** Abbreviations: PY, person-years; T2D, type 2 diabetes. *Adjusted for age (as a continuous variable), index year, co-morbidity score (no, low, or high), UICC stage (I–III), type of surgery (mastectomy with and without radiation therapy (RT), and BCS), neoadjuvant systemic therapy (yes, no), and baseline use (yes, no) of lipophilic statins, SSRI, and aspirin.

**Fig. 3:**
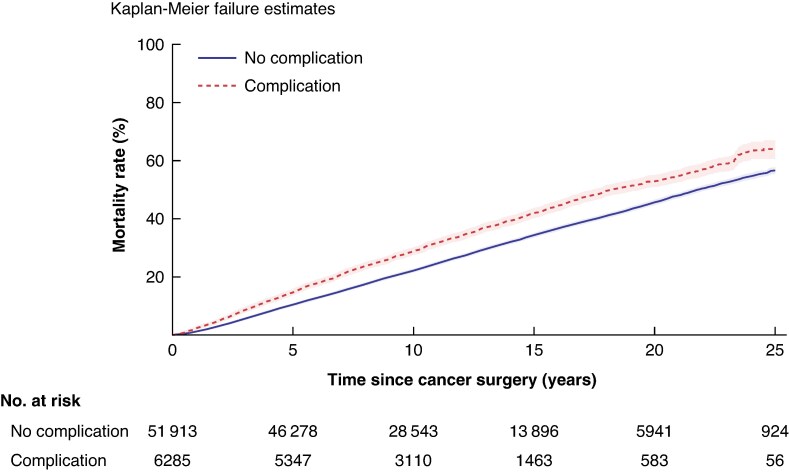
Cumulative all-cause mortality among 58 198 women with or without a postoperative complication following primary breast cancer surgery during 1996–2017

Among women who developed postoperative complications, 2418 died during a median follow-up of 9.9 years (i.q.r. 6.3–14.6), corresponding to an all-cause mortality rate of 35.8 (95% c.i. 34.4, 37.3) per 1000 PY (*Figs 2*, *3*). Among the 51 913 women without a complication, 16 661 deaths occurred in a median follow-up of 10.8 years (i.q.r. 7.0–15.5), corresponding to an all-cause mortality rate of 27.7 (95% c.i. 27.3, 28.1) per 1000 PY (*Figs 2*, *3*). Higher HRs of death among women who developed postoperative complications were observed when compared with those who did not have a complication (aHR = 1.11, 95% c.i. 1.06, 1.16) (*Fig. 2*).

Among the 26 193 10-year disease-free survivors, 516 and 4203 women with and without a postoperative complication died, corresponding to an all-cause mortality rate of 35.4 (95% c.i. 32.5, 38.6) and 29.2 (95% c.i. 28.4, 30.1) per 1000 PY respectively (*Fig. 2*). The aHR associating postoperative complications with all-cause mortality among the 10-year disease-free survivors was 1.05 (95% c.i. 0.95, 1.15) (*Fig. 2*).

### Type 2 diabetes and breast cancer outcomes

Overall, 2923 women had T2D at breast cancer diagnosis. Of these, 483 (16.5%) developed a postoperative complication. Women with T2D and complications were more often postmenopausal, had advanced breast cancer stage, more co-morbidity, and underwent mastectomy compared with T2D patients who did not develop complications (*Table 1*). Among 6285 women with postoperative complications, 53 with T2D and 694 without T2D developed early cancer recurrence (IR = 21.8, 95% c.i. 16.6, 28.5 and IR = 19.6, 95% c.i. 18.2, 21.1 per 1000 PY respectively). The interaction contrast was 2.5 (95% c.i. 2.2, 2.8) indicating minimal impact on the total recurrence rate of 11%. For late recurrence, the IRs were 24.0 (95% c.i. 21.5, 26.8) and 21.8 (95% c.i. 10.9, 43.7) respectively (interaction contrast in the late recurrence analysis was not calculated due to low numbers). T2D was not associated with higher adjusted hazard of early or late recurrence after postoperative complications (aHR_early_ = 1.10, 95% c.i. 0.81, 1.50 and aHR_late_ = 0.86, 95% c.i. 0.42, 1.79) (*Table 2*). Women with postoperative complications and T2D had higher risk of death compared with women without T2D (overall aHR = 1.50, 95% c.i. 1.30, 1.73; and in 10-year disease-free survivors aHR = 1.52, 95% c.i. 1.03, 2.24) (*Fig. 2*).

## Discussion

This study found no evidence of an association between postoperative complications following breast cancer surgery and the risk of early and late breast cancer recurrence. These findings did not vary by the presence of T2D at breast cancer surgery. In contrast, higher risk of all-cause mortality in women with postoperative complications was observed, particularly those with T2D.

The inconsistent findings on the association between postoperative complications following breast cancer surgery and breast cancer outcomes was exemplified in a recent systematic review including 10 cohort studies and 37 657 patients^31^. Contrasting with findings from the current study, some studies showed an increased risk of recurrence associated with postoperative complications^9,10^. However, the studies were limited by small sample size (Beecher *et al*.^9^ included 229 patients and Lee *et al*.^10^ included 438 patients) and restricted to women with wound complications after mastectomy and immediate breast reconstruction. None of the studies examined the impact of T2D on their findings. Still, stratified analyses in this study, investigating mastectomy and BCS separately, and restricting to surgical site complications only, did not show an association with recurrence risk.

Findings from this study are somewhat consistent with published research indicating negligible impact of complications on recurrence^32^ but a higher risk of all-cause mortality^33^ after surgical site complications. A previous study by Pedersen *et al.* included 30 711 women with a total of 205 926 PYs of follow-up and showed no increased risk of early breast cancer recurrence after reoperation for surgical bleeding^32^. The study did not, however, consider other postoperative complications. Complications other than surgical site complications were investigated in a recent study by Adwall *et al.* including 439 women with invasive breast cancer. They found that systemic infections following breast cancer surgery did not affect risk of local or distant recurrence^13^. This study enhances the current literature by incorporating surgical-site complications and a more systematic classification of postoperative complications. Furthermore, recent literature suggested a potential for delay to adjuvant cancer therapy due to postoperative complications following breast cancer surgery^34,35^. However, studies showed no difference in long-term outcomes among women with wound complications after mastectomy with immediate breast reconstruction or breast-conserving surgery despite a modest delay in initiation of adjuvant therapies^4,36^. Unfortunately, data on time to initiation of adjuvant treatment were not available in this study.

T2D is a well-established risk factor for all-cause death following most surgical procedures^37^. Analyses of late recurrence in the current study suggest that deaths among women with T2D are an important competing event. There were few 10-year disease-free breast cancer survivors with T2D in this study precluding the evaluation of any synergism between postoperative complications and T2D on long-term recurrence rates. For early recurrence, the interaction effect had minor impact on the association.

Several issues warrant consideration when interpreting these findings. A modified version of the late breast cancer recurrence algorithm was used as access to the CBC database was not possible. It would not be expected that this impacted the findings as the incidence of CBC is unlikely to be higher among women with a postoperative complication or T2D^38^. This study may be prone to selection bias. All women undergoing surgery with curative intent for breast cancer are treated according to national guidelines irrespective of T2D. Still, women with T2D may be less likely to receive adjuvant therapy due to the risk of side effects, and thus also more likely to experience recurrences. Nonetheless, no difference in recurrence risk was found in analyses stratified by T2D. Despite similarities between crude and adjusted estimates within primary analyses of recurrence, residual confounding may however persist. Information on BMI was not available in the data set and has been shown by others to associate with postoperative complications, T2D, and poorer breast cancer prognosis^17,39^.

This study showed that postoperative complications after primary breast cancer surgery were not associated with the risk of cancer recurrence but influences survival. No synergism between T2D and postoperative complications on the risk of recurrence was detected. The higher mortality rate associated with postoperative complications may reflect the underlying health of these patients, particularly among women with concurrent T2D. Future research may focus on investigating the systemic effect of postoperative complications to further optimize surgical and oncologic outcomes.

## Supplementary Material

znaf176_Supplementary_Data

## Data Availability

The data that support the findings of this study are available from Danish registries, but restrictions apply to the availability of these data, which were used under licence for the current study and so are not publicly available. Data are, however, available from the authors upon reasonable request and with permission.

## Meeting presentation

This study has been presented as a poster presentation at the ESMO Breast Annual Congress, Berlin, May 2025.

The paper is not based on previous communication to a society or meeting.
