# Supplementary material for: Impact of postoperative complications and type 2 diabetes on breast cancer recurrence and mortality
Source: Br J Surg. 2025 Aug 23;112(8):znaf176. doi: 10.1093/bjs/znaf176 (PMC12374189; doi:10.1093/bjs/znaf176)
Supplement: znaf176_Supplementary_Data [file znaf176_supplementary_data.docx]

**Impact of Postoperative Complications and Type 2 Diabetes on Breast Cancer Recurrence and Mortality**

Kasper A Kjærgaard, MD ^1,2^, Peer Christiansen, DMSc ^3^, Signe Borgquist, MD, PhD ^2,4^, Deirdre Cronin-Fenton, BSc, PhD ^1^

*^1^Department of Clinical Epidemiology, Department of Clinical Medicine, Aarhus University Hospital, Aarhus University, Aarhus, Denmark*

*^2^Department of Oncology, Department of Clinical Medicine, Aarhus University Hospital, Aarhus University, Aarhus, Denmark*

*^3^Department of Plastic and Breast Surgery, Department of Clinical Medicine, Aarhus University Hospital, Aarhus University, Aarhus, Denmark*

*^4^Department of Oncology, Clinical Sciences, Lund University, Sweden*

**Corresponding author.** Kasper Kjærgaard, MD, PhD **ORCID ID** 0000-0003-0614-7938; **Twitter** @KaKjaergaard

**Supplementary Materials - Index**

| **Supplementary Methods** |  |
| --- | --- |
| The DBCG registry | *page 2* |
| Late breast cancer recurrence algorithm  Interaction contrasts calculations | *page 2*  *page 3* |
| **Supplementary Figures and Tables** |  |
| Table S1 | *page 4* |
| Table S2  Figure S1 | *page 8*  *page 9* |
| **References** | *page 10* |
|  |  |

**Supplementary Methods**

*The DBCG registry*

The DBCG database was introduced in 1977 to improve prognosis in breast cancer and has since included comprehensive patient and clinicopathologic data on all women diagnosed with primary nonmetastatic breast cancer in Denmark.^1^ The completeness of the DBCG registration exceeds 95% each year.^2^

*Late breast cancer recurrence algorithm*

Codes included in the algorithm for late breast cancer recurrence:

- DNPR-registered metastases codes (ICD10 DC76-DC80)
- Pathology-registered SNOMED combinations. Combinations were 1) T code (*topography/location) in the breast with morphology codes M8 or M9 with 4 (direct spread), 6 (malignant metastases), 7 (local recurrence), or 9 (malignant tumor metastases or new primary tumor) in the fifth position (e.g., M8XXX4), 2) any T code (excluding the breast T codes) with morphology codes M8 or M9 with the numbers 6 or 9 in the fifth position.
- DNPR-registered cancer-directed treatment codes (SKS treatment codes) including radiotherapy (BWG), chemotherapy (BWHA), endocrine therapy (BWHC, BHHH, BOHJ13). Registration of these began in the DNPR in 2000.
- DNPR-registered surgical codes (according to the Danish Version of the Nordic Medico-Statistical Committee Classification of Surgical Procedures) including mastectomy (KHAC), breast conserving surgery (KHAB), and resection of the chest wall (KGAE16).
- A code specific for local breast cancer recurrence (ICD10 DC509X) or a code specific for recurrence surgery (according to the Danish Version of the Nordic Medico-Statistical Committee Classification of Surgical Procedures) (KHAF) in the DNPR. Registration of these began in the DNPR in 2012.

*Topography/location codes related to the breast:

T00100, T02424, T0242K, T0242L, T02430, T0243A, T0243B, T04000, T04010, T04020, T04030, T04100, T0410A, T0410B, T04200, T04202, T0420A, T0420B, T0420E, T0420F, T04280, T0428A, T0428B, T04400, T04441, T04442, T04600, T04800, T08350, T08352, T08353, T08354, T0835A, T0835B, T0835C, T0835D, T08710, T08711, T08712, T0871A, T0871B, T0871C, T08720, T08721, T08722, T09420, T0Y400, T0Y40A, T0Y40B, TY8101, TY8102, T08200, T08201, T08202, T0820A, T0820B, T0820C, T08210, T08211, T08212, T08220, T08221, T08222, T08280, T08281, T08282, T02400, T02401, T03400, TY062A, TY062B, TY123A, TY123B, TY1960, TY2960

*Example of early recurrence interaction contrasts calculations*

Baseline rate: IR_BC+NO COMPL_

Rate attributable to T2D alone: IR_BC+T2D & NO COMPL_ – IR_BC & NO COMPL_

Rate attributable to complications alone: IR_BC & COMPL_ – IR_BC & NO COMPL_

Rate attributable to interaction between T2D and complications:

IR_BC+T2D & COMPL_ – IR_BC & NO COMPL_ – (IR_BC+T2D & NO COMPL_ – IR_BC & NO COMPL_) – (IR_BC & COMPL_ – IR_BC & NO COMPL_)

**Supplementary Figures and Tables**

**Table S1** ICD-10 codes defining postoperative complications

| **Surgical complications** | **ICD-10 codes** |
| --- | --- |
| Post-surgical complication | T810, KHW |
| Reoperation due to complication | KTHA20, KTHA30, KTHA40, KTHW |
| Seroma | KQBA00 |
| Wound complication in the breast and/or axilla | T813, T814A, T814F, T814G, T814H, T819 |

**Bleeding**

| **Hospital-diagnosed spontaneous bleeding** | **ICD-10 codes** |
| --- | --- |
| Cerebral | I60, I61, I62 |
| Respiratory tract | R04.0, R04.2 |
| Upper gastrointestinal tract | I85.0, K25.0, K25.2, K25.4, K25.6, K26.0, K26.2, K26.4, K26.6, K27.0, K27.2, 27.4, K27.6, K28.0, K28.2, K28.4, K28.6, K29.0 |
| Lower gastrointestinal tract | K62.5, K92.0-K92.2 |
| Urinary tract | R31.9, N02 |
| Anemia from bleeding | D62 |

**Infections**

| **Hospital-diagnosed infectious diseases** | **ICD 10 codes** |
| --- | --- |
| Miscellaneous bacterial infections | A20-A38, A42-A44, A48-A49, A65A79 |
| Miscellaneous viral infections | A90-A99, B03-B09, B25-B34 |
| Candidiasis and other fungal infections | B35-B49 |
| Herpes simplex or zoster | B00-B02  (Additional codes- might overlap with codes in ‘B’ section: G05.1I, G05.1M, H03.1F, H13.1M, H19.0D, H19.2D, H19.2J, H22.0C, H62.1B, G53.0, G63.0F) |
| Tuberculosis | A15-A19 |
| Atypical mycobacteria | A31 |
| Bacteremia | A49.9, A39.4 |
| Sepsis | A40-A41, B37.7, A32.7, A54.8G, A02.1, A22.7, A26.7, A42.7, A28.2B |
| Abscess | A06.5, A54.1, B43, D73.3, E06.0A, E23.6A, E32.1, G06, G07, H00.0A, H05.0A, H44.0A, H60.0, J34.0A, J36, J38.3D, J38.7G, J39.0, J39.1, J39.8A, J85.1, J85.2, J85.3, K04.6, K04.7, K11.3, K12.2, K13.0A, K14.0A, K20.9A, K35.3A, K35.3B,  K57.0, K57.2, K57.4, K57.8, K61, K63.0, K65.0, K75.0, K81.0A, K85.8A, L02, L05.0, L05.9, M60.8A, M86.8A, M86.9A, N15.1, N34.0, N41.2, N45.0, N48.2, N49.2A, N61.9A, N61.9B, N70.0A, N70.0B, N71.0A, N73.0A, N73.0B, N73.2A,  N73.2B, N73.3A, N73.5A, N73.8A, N73.8C, N75.1, N76.4, N76.8A,  ***Except:*** A54.1B, B43.0, B43.8, B43.9, K57.0B, K57.0C, K57.2B, K57.2C, K57.4A, K65.0M, K65.0N, K65.0O, K65.0P |
| Skin infections | A46, H01.0, H03, H60.0, H60.1, H60.2, H60.3, H62, K12.2, K13.0, K61, M72.6, L01, L08 |
| Cellulitis | L03 |
| Other skin infections (including | J34.0, L00, L02, L04, L05, L06, L07, |
| carbuncle, furuncle, lymphadenitis, cutaneous abscess, cyst, and dermatitis) | L30.3, L73.8 |
| Central Nervous System infections (except meningococcal disease) | G00-G07, A80-A89 |
| Meningitis | G00, G01, G02, G03, A32.1, A39.0, A17.0, A20.3, A87, A54.8D, A02.2C, B37.5, B00.3, B01.0, B02.1, B05.1, B26.1, B38.4 |
| Gastrointestinal infections | A00-A09 |
| Intra-abdominal infection | K35, K37, K57.0, K57.2, K57.4, K57.8, K61, K63.0, K65.0, K65.9, K67, K75.0, K75.1, K80.0, K80.3, K80.4, K81.0, K81.9, K83.0, K85.9 |
| Viral hepatitis | B15-B19 |
| Heart infections(acute rheumatic fever, infectious pericarditis or myocarditis, endocarditis) | I00-I02, I30.1, I32.0, I33, I38, I40.0, I39.8, B37.6 |
| Upper respiratory tract infection | J00-J06, J36, J39.0, J39.1 |
| Influenza | J10-J11 |
| Pneumonia | J12-J18 |
| Other lower-respiratory tract infections | J20-J22, J44.0, J85.1, J86, J20-J22, J34.0, J35.0, J38.3C, J38.3D, J38.7B, J38.7F, J38.7G,  ***Except:*** J34.0E, J34.0F, J34.0G, J34.0H |
| Urinary tract infections | N10, N11, N12, N15.1, N15.9, N30, N33.0, N34, N39.0, N08.0, N13.6, N16.0, N28.8D, N28.8E, N28.8F, N29.0, N29.1,  ***Except:*** N30.1, N30.2, N30.4 |
| Female pelvic infections  (salpingo-oophritis, uterine | N70-N77 |
| infections, vulvovaginitis) |  |
| Septic arthritis, osteomyelitis, myositis | M00, M01, M86, M63.0, M63.2 |
| Infectious complications of procedures, catheters etc. | T80.2, T81.4, T82.6, T82.7, T83.5, T83.6, T84.5, T84.6, T84.7, T85.7, T88.0, T89.9  ***Except*:** T81.4A, T81.4F, T81.4G, T81.4H |
| Other infections or sequelae | B90-B99, K04.0, K05.2 |

**Venous Thromboembolism**

| **Hospital-diagnosed venous thromboembolism** | **ICD-10 codes** |
| --- | --- |
| Venous thromboembolism  (VTE)^1^ |  |
| Deep venous thrombosis (DVT) | I801-3 |
| Pulmonary embolism (PE)^2^ | I26 |
| Post-surgical thromboembolism | DT817B, DT817C |

**Arterial Cardiovascular Disease**

| **Hospital-diagnosed cardiovascular diseases** | **ICD-10 codes** |
| --- | --- |
| Acute myocardial infarction | I21 |
| Stent thrombosis | T823D, T823E |
| Angina pectoris |  |
| Stable angina pectoris | I20 (without I200), I251, I259 |
| Unstable angina pectoris | I200 |
| Heart failure | I500, I501, I502, I503, I508, I509, I110, I130, I132, I420, I426, I427, I428, I429 |
| Cardiac arrythmias |  |
| Atrial fibrillation or flutter | I48 |
| Bradycardia (sinus node dysfunction og AV-block) | I440, I441, I442, I443, I455A, I455B, I455C, I455G |
| Ventricular  tachycardia/fibrillation | I470, I472, I490 |
| Heart valve diseases |  |
| Mitralvalve-insufficiency and stenosis | I05, I34, I390, I511A |
| Aorticvalve-insufficiency and stenosis | I06, I35, I391 |
| Cardiac inflammation and infection |  |
| Endocarditis | I33, I38, I398 |
| Myocarditis | I40, I41, I090, I514 |
| Pericarditis | I30-132 |
| Aorta diseases |  |
| Aortadissection | I710 |
| Aneurisme/dilatation | I711-I716, I718-I719 |
| Claudicatio intermittens | I739A |
| Stroke |  |
| Combined ischemic + unspecified stroke | I63-I64 |
| Specified ischemic stroke | I63 |
| Unspecified stroke | I64 |
| Transient ischemic attack | G459 |

**Kidney Disease**

| **Hospital-diagnosed kidney diseases** | **ICD-10 codes** |
| --- | --- |
| Nephrotic syndrome | N04 |
| Glomerulonephritis (without nephrotic syndrome) | N00, N01, N03, N05 |
| Hypertensive nephropathy | I12, I13, I15.0, I15.1 |
| Chronic pyelonephritis/Interstitial nephritis | N11, N14, N15, N16 |
| Polycystic kidney disease | Q61.1-Q61.4 |
| Other and unknown chronic renal disease (none of those above, but): | N18-N19, N26, N27, N07, N08  (without N08.3) |

**Table S2** Incidence rates per 1000 person-years of early and late breast cancer recurrence according to postoperative complications and type 2 diabetes among women diagnosed with early-stage breast cancer from 1996 to 2017.

|  | Early recurrence | Late recurrence |
| --- | --- | --- |
|  | Incidence rate per 1000 PY  (95% CI) | Incidence rate per 1000 PY  (95% CI) |
| **Overall (complication within 30 days)**  No complication  Postoperative complication | 17.9 (17.4-18.4)  19.7 (18.4-21.2) | 26.0 (25.1-26.9)  23.9 (21.4-26.7) |
| **Overall**  No complication + T2D  Postoperative complication + T2D | 17.5 (15.4-19.9)  21.8 (16.6-28.5) | 34.0 (27.7-41.7)  21.8 (10.9-43.7) |

Abbreviations: CI, confidence interval; PY, person-years; T2D, type 2 diabetes

**Figure S1** Early and late breast cancer recurrence among women diagnosed with early-stage breast cancer from 1996 to 2017 with and without a postoperative complication, stratified by age groups, calendar period, comorbidity score, UICC stage, ER status, neoadjuvant therapy, type of surgery, and type of complication.


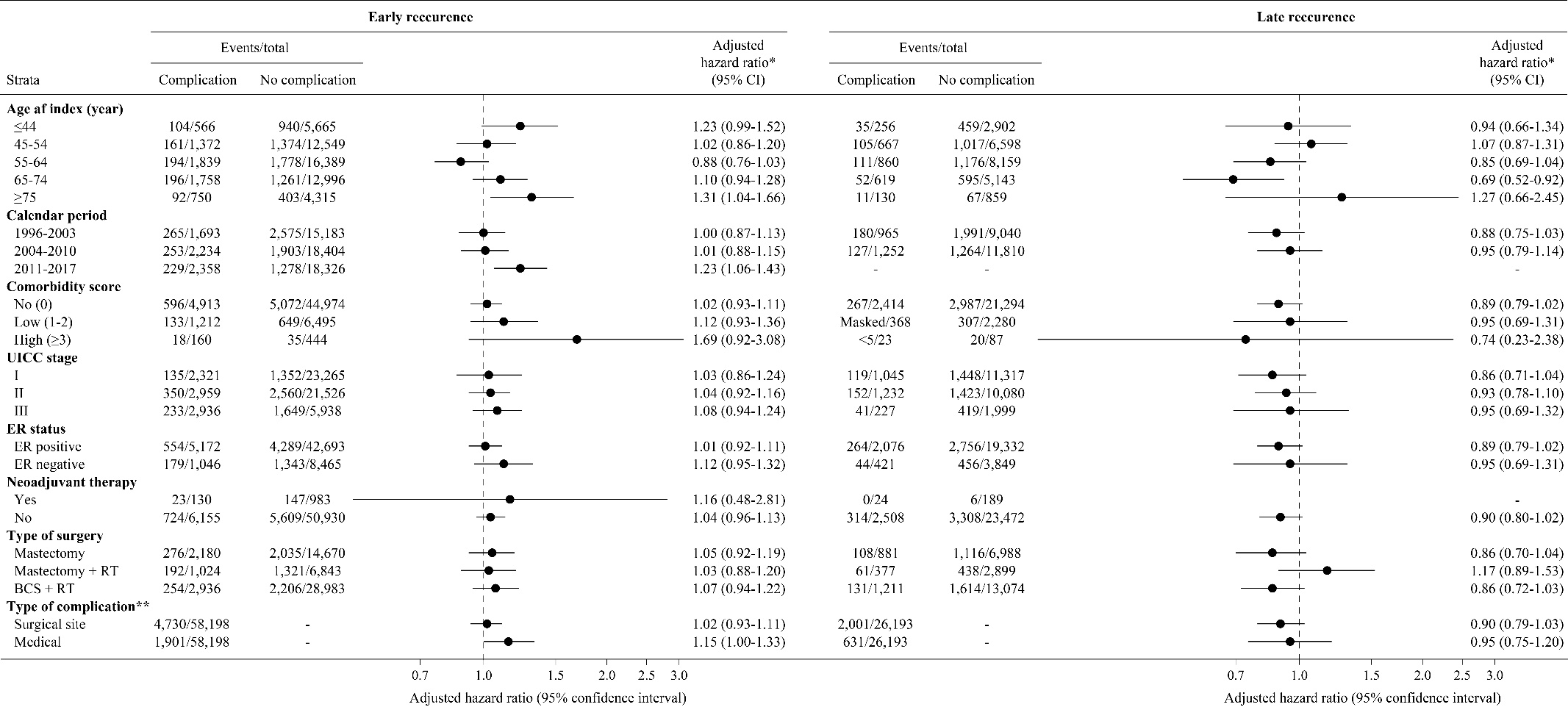


Abbreviations: BCS, breast-conserving surgery; CI, confidence interval; ER, estrogen receptor; RT, radiation therapy
*Adjusted for age (as a continuous variable), index year, comorbidity score (no, low, or high), UICC stage (I-III), type of surgery (mastectomy with and without RT, and BCS), neoadjuvant systemic therapy, and baseline use (yes, no) of lipophilic statins, SSRI, and aspirin.
**346 patients in the early recurrence cohort and 100 patients in the late recurrence cohort were classified as having a medical and surgical complication the same day and thus contributes with an event in both subgroups in the stratified analysis

**References**

1. Christiansen P, Ejlertsen B, Jensen M-B, Mouridsen H. Danish Breast Cancer Cooperative Group. *Clinical Epidemiology*. 2016;Volume 8:445-449. doi:10.2147/clep.s99457

2. Group TDBCC. Kvalitetsdatabase for Brystkræft - National Årsrapport 2020. 2020;
